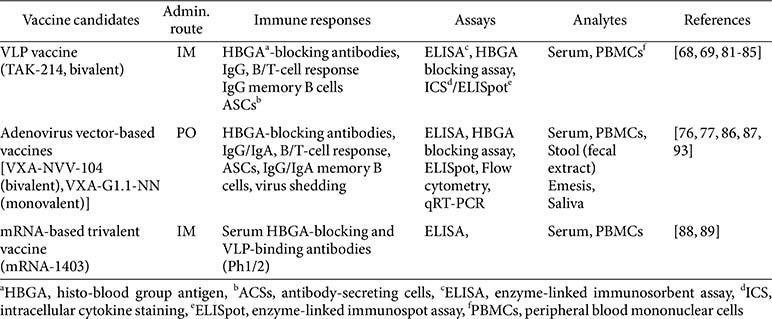# Current Perspectives and Future Directions in the Immunogenicity Landscape of Norovirus Vaccines

**DOI:** 10.4014/jmb.2509.09003

**Published:** 2025-11-10

**Authors:** Hee-Jung Lee, Doyoung Yoon, Haewon Jung, Seo-Yun Hong, Young Bong Kim, Jong-Won Oh

**Affiliations:** 1 Department of Biomedical Science and Engineering, Konkuk University, Seoul 05029, Republic of Korea; 2 Department of Biotechnology, Yonsei University, Seoul 03722, Republic of Korea; 3 Graduate Program in Bio-industrial Engineering, Yonsei University, Seoul 03722, Republic of Korea

**Keywords:** Human norovirus, vaccine, efficacy evaluation, immunogenicity assessment

## Abstract

Human norovirus (HuNoV) is a leading cause of acute gastroenteritis worldwide, causing severe illness and death in vulnerable populations, including infants and the elderly. Despite advances in norovirus vaccine candidates such as virus-like particles (VLPs), adenovirus-based oral vaccines, and mRNA vaccines, no vaccine has been approved yet. Current clinical trials primarily target the GI.1 and GII.4 genotypes responsible for most outbreaks. However, the extensive genetic diversity of HuNoV, along with continual antigenic evolution, poses significant challenges for developing broadly protective vaccines. Recent advances in experimental tools, including human intestinal enteroid cultures and surrogate neutralization assays, have improved norovirus vaccine efficacy assessment. Nevertheless, the lack of robust culture systems and animal models that faithfully mimic human infection continues to limit comprehensive evaluation of immune responses to diverse variants. Moreover, standardized correlates of protection, particularly those addressing mucosal immunity critical for infection prevention, remain to be established. This review integrates current immunogenicity assessment methodologies and evaluates ongoing HuNoV vaccine strategies, with emphasis on variant strain selection and platform technologies. We discuss key challenges related to population diversity, immune imprinting, and the complex interplay between systemic and mucosal immune responses as influenced by vaccine delivery routes and adjuvant formulations. By integrating recent advances in vaccine platforms, immunological tools, and delivery strategies, this review provides a framework for addressing critical obstacles in norovirus vaccine development. Such integrative perspectives are crucial for developing safe, effective, and broadly protective vaccines that offer meaningful benefits to global health.

## Introduction

Human norovirus (HuNoV) is a leading cause of acute viral gastroenteritis worldwide, accounting for approximately 685 million total cases annually [1-3]. The virus was first identified following an outbreak of gastroenteritis in Norwalk, Ohio, USA, in 1968 and subsequently named the Norwalk virus in 1972 [4]. Advances in molecular biology, such as gene cloning and sequencing, later revealed that norovirus, along with other related viruses, belongs to the *Caliciviridae* family, sharing a similar genome organization [5].

**Fig. 1:**
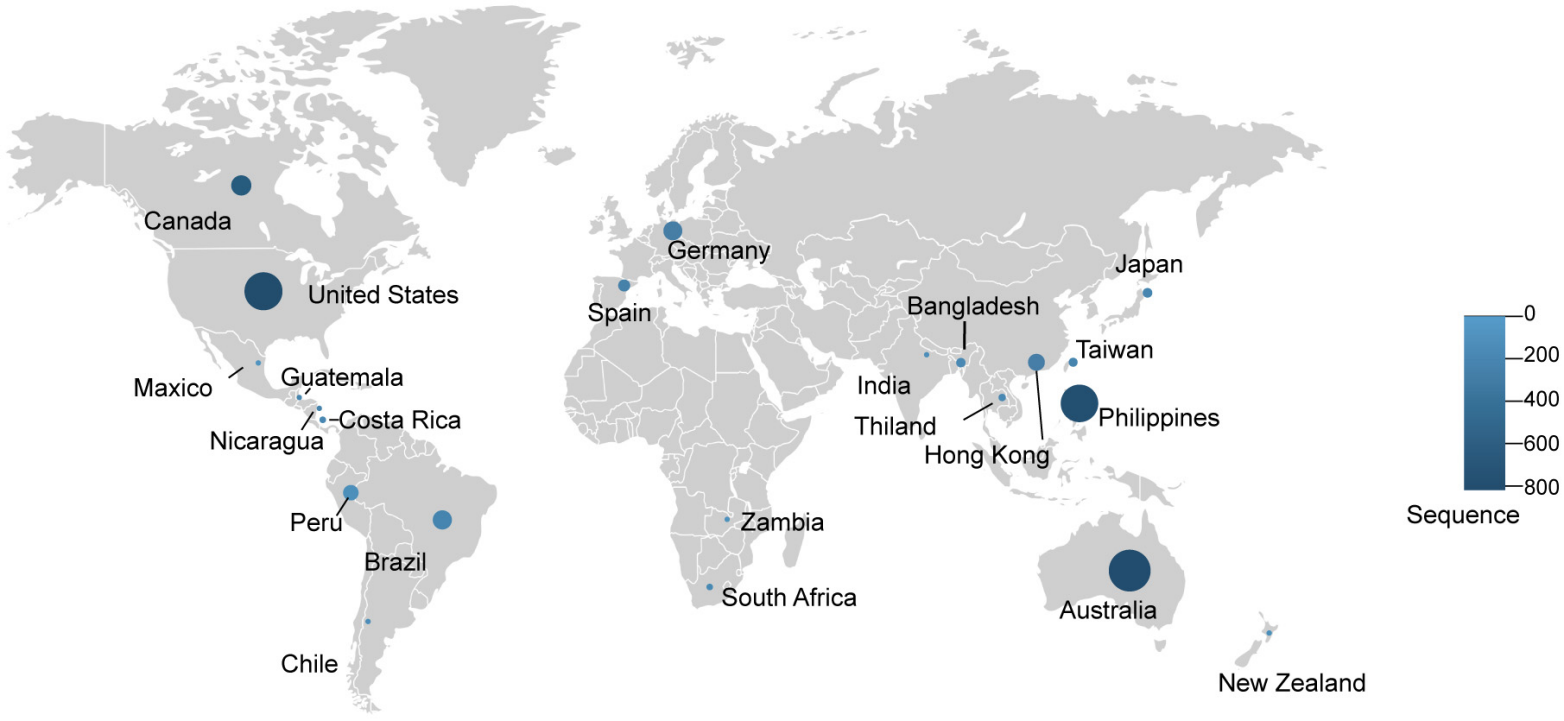
Widespread occurrence of norovirus infection across continents (2015–2025). The map illustrates the number of genetic sequences of norovirus strains identified in children under 5 years of age who were hospitalized with acute gastroenteritis. The data were collected from 22 countries participating in the NoroSurv global surveillance network [9] between December 2015 and July 2025. Circle sizes are proportional to the number of reported sequences, with a minimum size for countries reporting fewer than 100. The circle color uses a blue gradient, where lighter shades indicate lower counts and darker shades indicate higher counts.

Norovirus is highly contagious, readily transmitted through contaminated food or water, direct contact with infected individuals, or exposure to contaminated surfaces [1]. Its low infectious dose and environmental persistence facilitate rapid spread, especially in communal settings such as schools, hospitals, and long-term care facilities, making outbreaks difficult to control [2, 3]. As a result, norovirus imposes a substantial global health and economic burden [4-6]. Human norovirus infects people of all ages, but it has the greatest impact on young children and the elderly. Both groups are more susceptible to severe symptoms, complications, hospitalizations, and, in rare cases, death. Each year, there are an estimated 685 million total cases of acute gastroenteritis caused by norovirus, of which 200 million cases of norovirus occur worldwide among children under 5 (Fig. 1), leading to about 50,000 child deaths, with the vast majority occurring in developing and low-income countries [7]. By age 5, approximately 1 in 40 children will visit an emergency room, and 1 in 160 will be hospitalized due to norovirus illness. Most norovirus-associated hospitalizations and deaths (136,000 to 278,000 deaths worldwide annually) occur among adults aged 65 years and older. Elderly adults not only experience more severe disease but also have longer symptom duration and a higher risk of complications like dehydration, sepsis, or cardiac events, especially if they have underlying chronic conditions [1, 8]

Recognizing these challenges, the World Health Organization (WHO) highlighted in 2019 the urgent need for preventive measures, including vaccines and antivirals, to combat norovirus infections [10, 11]. Despite substantial research efforts, no licensed human norovirus vaccine is currently available, though several candidates are in clinical trials [3, 12-14]. Norovirus vaccine development faces significant obstacles, notably the extensive genetic diversity of the virus, the lack of sustained natural immunity following infection, and the absence of robust and convenient cell-culture systems and animal models for preclinical evaluation [15, 16].

This review offers a comprehensive and updated overview of recent advances, challenges, and future directions in human norovirus vaccine development. We summarize current vaccine candidates and critically examine the methodologies used to assess their immunogenicity. We review both traditional and innovative immunological assays, emphasizing advances as well as ongoing challenges in assessing vaccine-triggered immune responses. By integrating these perspectives, the review provides insights for supporting the development of the next generation of norovirus vaccines.

## Genome Organization, Transmission Dynamics, and Cellular Entry of Norovirus

Norovirus belongs to the *Caliciviridae* family and is characterized by a non-enveloped, icosahedral virion measuring 27–40 nm in diameter [17-19]. The virus possesses a single-stranded, positive-sense RNA genome approximately 7.5 kilobases (kb) in length. This genome is organized into three open reading frames (ORFs). ORF1 encodes nonstructural proteins (NS1/2, NS3, NS4, NS5, NS6, and NS7) required for viral replication and post-translational processing [20-22]. ORF2 encodes the major capsid protein VP1, while ORF3 produces the minor structural protein VP2, which is thought to contribute to capsid stability and virion maturation [23, 24].

Noroviruses are highly contagious, primarily due to their exceptional environmental stability [2]. The non-enveloped structure confers resistance to a broad range of temperatures, acidic pH, and common chemical disinfectants, including chlorine-based agents [25]. This robustness enables the virus to persist on contaminated food, water, and fomites, making fecal-oral transmission the predominant route of infection [26, 27]. Remarkably, norovirus is infectious at extremely low doses, as few as 10–100 viral particles are sufficient to cause illness [28-30]. Moreover, infected individuals, including those without symptoms or during convalescence, can shed substantial quantities of virus, increasing the likelihood of outbreaks, particularly in confined environments such as schools, hospitals, and cruise ships [28, 31]. Vomiting induced by infection can generate virus-laden aerosols, facilitating short-range airborne transmission and secondary contamination of surfaces, which may serve as sources for indirect, contact-mediated spread [32-35].

The initial step of norovirus infection involves attachment to histo-blood group antigens (HBGAs), carbohydrate molecules expressed on the surface of intestinal epithelial cells [36, 37]. This interaction is mediated by the P2 subdomain of the VP1 capsid protein, which binds specific HBGA structures in a genotype-dependent manner [38]. Following the attachment, the virion is internalized via endocytosis, while subsequent capsid disassembly and the release of viral RNA into the cytoplasm initiate replication [39]. Host susceptibility to norovirus is largely determined by genetic variation in HBGA expression. The FUT2 gene encodes fucosyltransferase 2, an enzyme essential for HBGA synthesis [40-42]. Non-secretor individuals, who lack the functional FUT2 gene and therefore do not express HBGAs on intestinal tissues, are generally resistant to infection by prevalent genotypes such as GI.1 and GII.4 [43]. In contrast, secretor-positive individuals, who express functional HBGA receptors, are susceptible to a broad spectrum of norovirus strains [36]. This genetic heterogeneity among individuals not only influences their susceptibility to infection risk but also modulates population-level outbreak dynamics and impacts the vaccine efficacy [44].

## Challenges to the Human Norovirus Vaccine Development

Despite the significant global disease burden caused by HuNoV, no licensed vaccines are yet available. Vaccine development is hindered by several technical obstacles, including widespread genetic diversity, the lack of robust cell culture systems for viral propagation, uncertain immune correlates of protection, and limited animal models that faithfully recapitulate human infection.

### Genetic and Antigenic Diversity

**Fig. 2:**
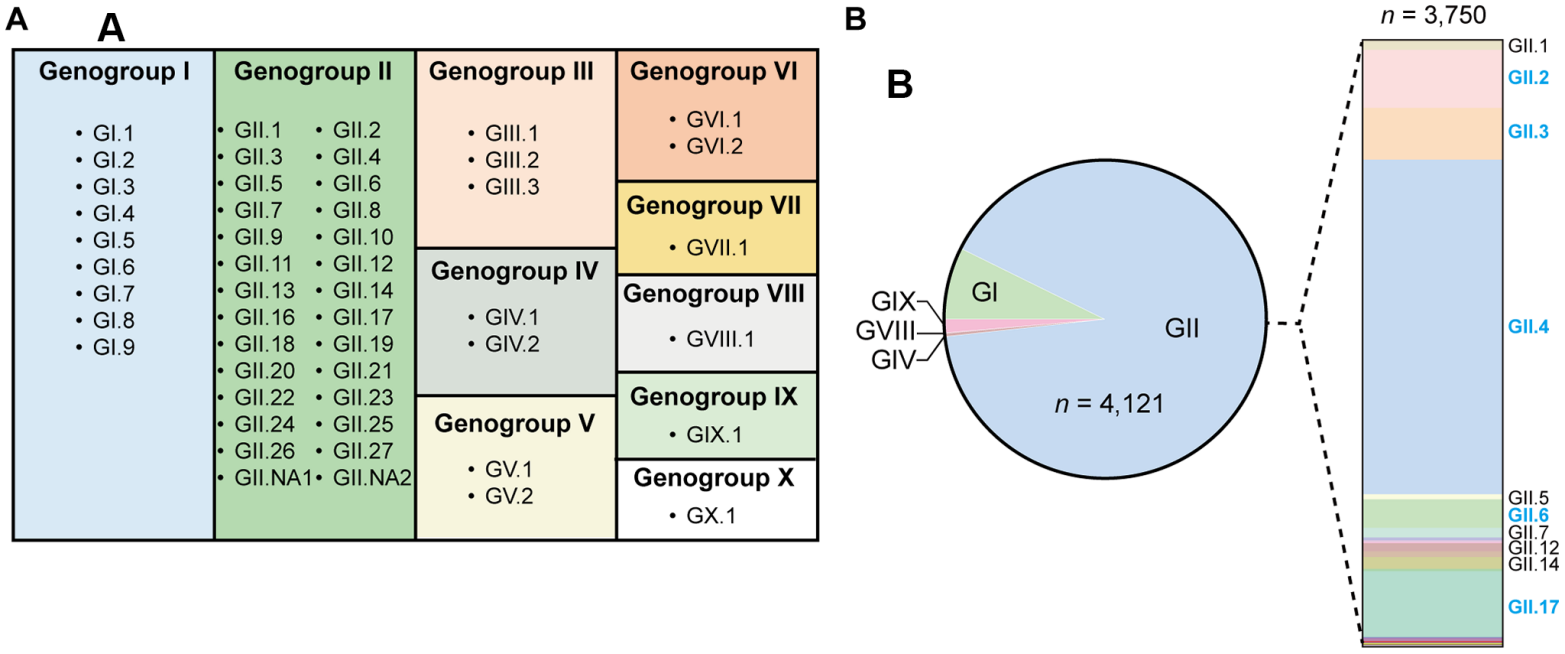
Genetic diversity of NoVs. (**A**) Schematic overview of norovirus classification showing the ten recognized genogroups (GI–GX) and their respective genotypes. (**B**) Distribution of human-infecting norovirus genogroups (GI, GII, GIV, GVIII, and GIX) with the five most prevalent GII-associated genotypes (GII.2, GII.3, GII.4, GII.6, and GII.17) highlighted in blue, demonstrating their importance in human norovirus disease burden and public health impact.

The extraordinary genetic diversity of noroviruses has long been recognized as a major challenge for vaccine design. In 2002, following the rapid expansion of known norovirus strains, the International Committee on Taxonomy of Viruses (ICTV) formally established the genus Norovirus, with detailed taxonomic classification driven by sequence analysis of viral proteins [45, 46]. Noroviruses are now classified into ten genogroups (GI–GX) based on sequence divergence in the major capsid protein (VP1) and the RNA-dependent RNA polymerase (RdRp). Within these genogroups, 49 recognized genotypes [9 GI, 27 GII, 3 GIII, 2 GIV, 2 GV, 2 GVI, and one genotype each for GVII, GVIII, GIX (formerly GII.15), and GX] have been identified [17] (Fig. 2). Genogroups GI and GII are primarily responsible for human infections and are therefore considered the primary targets for vaccine development [47, 48]. The high genetic diversity of noroviruses underpins their ability to cause frequent outbreaks and evade immunity.

Notably, VP1 exhibits significant variability, not only between genogroups but also among numerous genotypes, especially within GII, which includes epidemiologically important variants such as GII.1, GII.3, GII.4, and GII.17, as well as variants of the GII.4 genotype.

A recent report highlights a shift in norovirus outbreaks in the United States, where GII.17 rose from less than 10% of outbreaks in the 2022–23 season to 75% in the 2024–25 season, surpassing the previously dominant GII.4 strain [49]. Additionally, outbreaks caused by GII.17 have increased in several European countries, including Austria, Germany, France, Ireland, the Netherlands, and England, with prevalence ranging between 17% and 64%[50]. In pediatric patients from Beijing, China, GII.17 was the predominant genotype in 2024 (41.43%), followed by GII.4 Sydney (34.29%) and GII.3 (20.0%) [51]. Similarly, GII.17 has emerged as the predominant strain in South Korea, as reported in a 2024 wastewater-based epidemiology study [52]. Whether this rapid expansion of GII.17 will influence other regions and maintain its dominance remains to be seen, emphasizing the need for continued surveillance. Concurrently, studies investigating immune escape by GII.17 from GII.4-specific antibodies are needed to predict the persistence of GII.17 as a dominant genotype and to design vaccines that maintain efficacy and comprehensive protection against emerging GII.17 variants.

As such, this antigenic heterogeneity complicates the development of a broadly protective vaccine, as immune responses generated against one strain often display limited cross-reactivity to others. As a result, rational vaccine design must rely on careful selection of representative strains, informed by global surveillance data. Furthermore, the need for frequent updates to vaccine formulations, similar to the annual updates for influenza vaccines and the frequent updates of COVID-19 vaccines, may be necessary to address the dynamic evolution and shifting dominance of norovirus strains in the human population.

### Cell Culture Systems

A major obstacle in norovirus research has been the inability to efficiently propagate HuNoV in conventional monolayer cell cultures. This limitation has historically hampered studies of viral biology, immunogenicity, and the evaluation of candidate vaccines and antivirals.

In 2017, a significant step forward was made with the demonstration that GII.4 Sydney, a pandemic norovirus strain, could replicate in the human B lymphoblastoid cell line BJAB. However, this system exhibits narrow strain specificity, supporting replication of only certain genotypes (notably GII.4 and GII.6) [53, 54]. Furthermore, B cells lack the polarity and immunological characteristics of intestinal epithelial cells, which are the natural site of norovirus infection, and produce relatively low viral yields (typically a 10- to 50-fold amplification) with variable reproducibility between laboratories [54]. These limitations render B-cell-based systems inadequate for generating the high viral titers needed for robust immunological and virological studies.

A transformative advance was made with the development of human intestinal organoids (HIOs), which accurately recapitulate the complex architecture and cellular diversity of the human gut. HIOs have been shown to support the replication of GI.1 and 11 different GII genotypes, including clinically relevant strains such as GII.4 and GII.17, thereby overcoming the limitations of traditional 2D cell culture [55, 56]. CRISPR-engineered human intestinal enteroids (HIEs) are another advanced model that allows for precise genetic modifications, enabling a more accurate replication of human intestinal physiology and potentially enhancing the efficiency of HuNoV cultivation [57].

In addition to culture systems, extensive efforts over the past three decades have focused on establishing reverse genetics tools for HuNoV and mouse norovirus [58, 59]. Given the genetic heterogeneity of clinical isolates and biosafety considerations, homogeneous recombinant noroviruses rescued from infectious cDNA clones would be invaluable for standardized evaluation of vaccine candidates and antiviral agents for HuNoV. Such systems hold promise for overcoming the remaining barriers to robust HuNoV propagation, thereby accelerating the development of vaccines and therapeutics.

### Animal Models

Researchers have explored several norovirus infection animal models that recapitulate key aspects of human infection, immune responses, and disease pathogenesis [60]. Although establishing reliable models has remained challenging, each model offers distinct advantages and limitations.

Gnotobiotic (Gn) pigs are recognized as an excellent animal model for human norovirus [61, 62]. Gn piglets demonstrated susceptibility to oral infection by human norovirus GII.4 strains, as evidenced by the detection of viral RNA in feces, intestinal lesions, active viral replication in the gut, and relevant antigen responses. They display disease progression that closely mirrors that observed in humans, including mild diarrhea and viral shedding patterns [63]. These features make them highly valuable for studies on pathogenesis, immunity, and vaccine efficacy.

Gn calves have been shown to support replication and induce enteropathogenic effects upon infection with the human norovirus strain GII.4-HS66 [64]. Experiments confirmed successful virus replication, shedding in feces, diarrhea, and localized immune responses in the intestine, closely resembling the disease seen in human cases

Chimpanzees have proven useful as a model for studying norovirus replication and host immunity [65]. When inoculated with human norovirus, chimpanzees exhibited virus shedding and developed serum antibody responses similar to those observed in humans, though typically without clinical symptoms. Viruses were detected in the jejunum and duodenum. Notably, a detectable level of virus was found in the liver, whereas no detectable level of viremia was observed, necessitating further investigation into this finding to elucidate its biological implications. Virus-like particles (VLPs) administered to chimpanzees induced protective immunity, although the virus was delivered IV for the challenge experiment, providing an effective model to explore vaccine efficacy.

Despite their physiological relevance, germ-free animals like Gn pigs or calves, and non-human primates come with significant practical limitations: (1) high cost of maintenance and experimentation in these animals, (2) ethical concerns, and (3) limited accessibility. These constraints limit their application in broad vaccine development efforts [66]. Recent advances in genetic engineering have produced BALB/c Rag-γc-deficient mice, the first manipulable rodent model for human norovirus [66, 67]. A notable breakthrough is the development of immunocompromised mice engrafted with human intestinal organoids derived from H9 human embryonic stem cells (H9tHIE), which efficiently support human norovirus replication *in vivo* and provide a physiologically relevant platform for studying viral biology [57]. While these mice lack functional immunity, they are an important step toward models that better reflect human norovirus infection.

## Representative Norovirus Vaccines and Assays for Evaluation of Their Efficacy

Current HuNoV vaccine development pipelines emphasize the engineering of multivalent immunogens to broaden epitope coverage across diverse genotypes, coupled with the rational selection and optimization of high-potency immunostimulatory adjuvants, particularly for VLP and recombinant subunit formulations, to drive sustained humoral and mucosal immunity with durable memory B- and T-cell responses. Investigational platforms include VLP-based constructs, recombinant subunit vaccines, viral vector-mediated delivery systems, and lipid nanoparticle (LNP)-encapsulated mRNA modalities (Table 1).

A variety of HuNoV vaccine candidates have been developed by major pharmaceutical companies, including Hille Vax/Takeda Pharmaceuticals, Vaxart, Moderna, and research institutes in China (Chengdu Kanghua Biological Products, Anhui Zhifei Longcom Biopharmaceutical, and Shanghai Institute of Immunity and Infection), Europe (Icon Genetics GmbH), and South Korea (InThera and RpexBio). Among the various candidates, VLP-based vaccines represent the most clinically advanced approach to human norovirus immunization, with several candidates, such as Takeda’s TAK-214, reaching late-stage (Phase 2b) clinical trials. In parallel, oral vaccine and mRNA-based vaccine platforms are advancing rapidly, driven by their potential to elicit broad and durable immune protection. Oral vaccines can directly stimulate mucosal immunity, which is critical for defense against enteric viruses like norovirus, while mRNA vaccines offer rapid design flexibility to address viral diversity and emerging strains. Together, these approaches are gaining attention for their capacity to induce both systemic and, in certain delivery formats, mucosal immune responses, which will support more comprehensive norovirus protection.

The primary efficacy evaluation methods used in representative norovirus vaccines in preclinical or clinical trials include assays for humoral and cellular immune responses, aiming to provide correlates of immune responses. Importantly, mucosal immune responses differ depending on the administration route of individual vaccine candidates. Here, we summarize the key assays used to test the efficacy of three representative HuNoV vaccine candidates, employing different platforms, in preclinical and clinical studies (Table 2).

### VLP and Subunit Vaccines

TAK-214 (HIL-214) represents the most advanced norovirus vaccine platform, currently in Phase 2b clinical evaluation. It is formulated to target GI.1 and GII.4 genotypes with aluminum hydroxide as an adjuvant. The Phase 2b NOR-211 trial (2024), conducted among U.S. Navy recruits, demonstrated significant efficacy in preventing moderate-to-severe gastroenteritis [68]. The results indicated that HBGA-blocking antibody titers show a robust correlation with protection in clinical settings.

In a later study, the same bivalent vaccine, generating strong homotypic (matched genotype) neutralizing antibody responses as expected, could confer protection against non-vaccine genotypes, suggesting that cellular and/or other antibody-mediated mechanisms may contribute to cross-protection [81]. This study suggested that the choice of antibody assay impacts the assessment of norovirus vaccine immunogenicity, with HBGA-blocking and neutralizing antibodies closely correlated within a given genotype.

Subunit vaccine development is primarily focused on the GII.4 VP1 protruding (P) domain. The domain can self-assemble into multimeric complexes, known as P particles, when expressed in systems such as *E. coli*. While smaller than VLPs, these retain receptor-binding and immunologic properties, generating high levels of antigen-specific IgG in animal models and stimulating CD4^+^ T cell responses. In mice, the P domain, with a cysteine-containing tag at its end, formed the P particles efficiently and induced a neutralizing antibody that blocked NoV VLP binding to the HBGA receptors [90, 91].

Although neither intranasal (IN) nor intramuscular (IM) administration of a VLP vaccine has been reported to induce fecal IgA, an oral VLP vaccine elicited a 4-fold average increase in fecal IgA, with 3 out of 10 vaccinees showing a 3-fold increase in antigen-specific IgA [92]. However, these IgA responses were substantially lower, approximately 6- to 12-fold, than those induced by an adenovirus vector-based oral vaccine [77]. These previous findings underscore the importance of selecting a suitable vaccine platform to optimize mucosal immune responses and ultimately enhance vaccine efficacy.

### Viral Vector-Based Oral Vaccine

Oral vaccines are gaining attention due to their ability to induce robust mucosal immunity, which is crucial for pathogens like norovirus. Vaxart’s VXA-G1.1-NN, delivered as an oral tablet, completed a Phase 1 trial involving 66 healthy subjects. This monovalent vaccine is based on a recombinant non-replicating adenovirus type 5 encoding VP1 from the GI.1 Norwalk strain of norovirus and a double-stranded RNA (dsRNA) as an adjuvant. Notably, the vaccine induced α4β7^+^ IgA plasmablasts, a specialized subset of IgA-secreting cells that express the mucosal-homing integrin α4β7, enabling them to migrate to the intestinal mucosa for local immune defense. VP1-specific IgA antibodies increased in saliva by day 28 post-vaccination and persisted in fecal samples up to day 180, demonstrating relatively durable mucosal immunity [77].

In a Phase 1b trial in older adults aged 55–80 years, the vaccine demonstrated sustained mucosal immunity lasting up to 210 days, confirming its capability to induce gut-targeted mucosal immune responses even in an age group known for diminished vaccine responsiveness [76].

Further, a Phase 2 placebo-controlled human challenge study in healthy adults aged 18 to 49 years showed that the oral vaccine induced robust mucosal and systemic immune responses, marked by significant increases in mucosal (in saliva, nasal lining fluid, and stool) IgA and serum IgG levels, as measured by ELISA [93]. The vaccine reduced norovirus infection rates by 30%, lowered the incidence of gastroenteritis, and decreased viral shedding in stool and vomit. Overall, the vaccine was well-tolerated and safe across all doses. Notably, machine learning analyses identified serum functional HBGA-blocking antibody and fecal IgA as strong correlates of protection [93].

Despite the promise of mucosal vaccine delivery for norovirus, several challenges remain. While oral delivery of viral vector-based vaccines can effectively mount local mucosal immunity to prevent viral entry and shedding in the gut, concerns about the longevity of immune responses persist. Intranasal delivery faces unique hurdles, such as potential safety concerns regarding vaccine access to the brain via olfactory pathways and difficulties in achieving long-lasting protective immunity [94, 95].

Moreover, studies on rotavirus and other vaccines have highlighted the pivotal role of the gut microbiome in modulating vaccine-induced immune responses. Therefore, variability in gut microbiota composition may influence vaccine efficacy, making this an important area for ongoing investigation [96, 97].

### mRNA-Based Vaccine

Building on the success of COVID-19 vaccines, Moderna’s mRNA-1403, a trivalent NoV vaccine, is currently progressing through Phase 3 clinical trials (NCT06592794) [88]. The vaccine represents a promising next-generation approach, leveraging mRNA technology well-suited for multivalent formulations. In a Phase 1/2 trial, the trivalent mRNA vaccine mRNA-143 (targeting GII.4, GII3, and GI.3 genotypes) elicited robust serum HBGA-blocking antibodies and binding antibodies against vaccine-matched NoV genotypes at 1 month post-vaccination [88, 89]. Among both younger adults (18–59 years of age) and older adults (60–80 years of age), a single injection induced serum HBGA-blocking antibody titers with a geometric mean ratio (GMR) of approximately 64 against GII.4 across all dose levels (low, medium, high). The Pan-Ig VLP-binding titers were comparable across age groups and doses, with a geometric mean titer of around 10^6^. While no major safety concerns were observed in earlier Phases (1 and 2), the Phase 3 clinical trial of mRNA-1403 was placed on hold by the US FDA in February 2025 due to a single reported case of Guillain-Barré syndrome (GBS) in a participant [98]. GBS has been reported as a rare adverse event following various viral vaccines, including influenza vaccines, with a higher risk of GBS associated with SCoV2 and influenza virus infections than with vaccinations [99]. It is anticipated that the trial will resume once the FDA completes its assessment and any additional required measures are implemented.

Mucosal antibody responses, including secretory IgA (sIgA), have been shown to be enhanced by vaccination in individuals with prior infection, suggesting that mRNA vaccination may stimulate mucosal immunity primed by natural SARS-CoV-2 exposure [100]. Similarly, the widespread prevalence of norovirus exposure presents an important immunological opportunity. Since the vast majority of individuals over 5 years old have encountered norovirus previously, parenterally administered vaccines may effectively reactivate existing immune memory. It is reasonable to speculate that norovirus mRNA vaccines could recall memory T and B cells residing in gut-associated lymphoid tissue (GALT) and secondary lymphoid organs, including the spleen. Upon activation, these memory cells may drive sIgA production at mucosal surfaces in the distal ileum, potentially providing protection against viral entry. Notably, public data on mucosal IgA responses following mRNA-1403 vaccination have not yet been disclosed.

Furthermore, a bivalent mRNA norovirus vaccine encoding VP1 antigens from GI.1 and GII.4 genotypes has demonstrated strong humoral and cellular immune responses in preclinical studies [79]. These include high-titer HBGA-blocking antibodies and activation of CD4^+^ and CD8^+^ T cells. Sera from vaccinated mice, diluted 1,000-fold, effectively protected human intestinal enteroids from GII.4 infection, reducing viral replication by over 90%.

Future investigations should assess whether this bivalent vaccine confers cross-protection against other globally circulating variants. Additionally, studies are warranted to determine whether intramuscular administration of HuNoV mRNA vaccines can enhance memory T and B cell responses, particularly in individuals likely to be seropositive for GII.4, and whether these vaccines can induce mucosal sIgA production in the gut.

## Humoral Immune Responses to Norovirus Infection and Vaccination

Humoral immunity is crucial for preventing norovirus infection, as it generates antibodies that target the virus’s entry mechanisms. Depending on the routes of immunization in experimental animals and during clinical trials, systemically circulating antibodies, such as IgG and IgA in sera, and antibodies from mucosal immunity, such as sIgA in salivary, nasal washes, feces, or rectal swabs, can be monitored to evaluate vaccine efficacy and provide proper correlates of immunization (COI).

Higher serum antibody levels have been shown to associate with protection from norovirus infection [101, 102]. In addition, elevated serum IgA levels after infection or vaccination are associated with higher levels of mucosal IgA and blocking antibodies. Norovirus-specific IgA antibodies are crucial for protection; however, it is the mucosal sIgA, not serum IgA per se, that directly blocks norovirus in the gut. A previous study demonstrated that IgA antibodies, especially dimeric forms, show higher HBGA blocking potency than IgG, consistent with the multimeric IgA structure providing increased avidity and functional capacity [103]. The authors also found that dimeric IgAs had superior blocking potency compared to monomeric IgA. Nevertheless, serum IgA levels often reflect or correlate with mucosal and salivary IgA concentrations.

Despite the importance of mucosal immunity, its short duration, the short half-life of sIgA, oral tolerance to antigens, and the degradation of these proteins in a harsh stomach environment impede the translation of oral norovirus vaccines to practice. Reflecting this and other features of each vaccine platform, more non-oral VLP-based and mRNA-based vaccines, which are delivered via IM injection, are under development and currently at the later stages of clinical trials. These vaccines primarily increase IgG levels in sera, and these antigen-binding and neutralization IgG levels in systemic circulation were shown to be a reliable COI for the vaccines conferring protective immunity in clinical studies [74].

In summary, high levels of both dimeric mucosal sIgA and monomeric serum IgA are indicative of effective norovirus vaccines. Additionally, antigen-specific IgG and neutralizing IgG antibody levels in sera, which persist for a longer period (> 6 months), are widely used as a COI in evaluating norovirus vaccines administered via IM or SC routes.

The following sections provide an overview of key humoral immunological assessment parameters essential for evaluating vaccine immunogenicity and efficacy. These include quantification of antigen-specific IgG and IgA antibodies, as well as functional evaluation of blocking antibodies, specifically HBGA-blocking, porcine gastric mucin (PGM)-blocking, and virus-entry-blocking neutralization antibodies.

### Assessment of Antigen-Binding Antibody Titer

The enzyme-linked immunosorbent assay (ELISA) remains the gold standard for quantifying norovirus-specific IgG and IgA antibodies in serum, providing a robust measure of systemic immune responses following vaccination. In addition, sIgA titers in saliva and feces reflect mucosal immunity, a critical yet often under-measured component of protection against norovirus. sIgA is the predominant immunoglobulin on mucosal surfaces and serves as a crucial barrier against norovirus infection by directly blocking viral attachment and entry into epithelial cells [76, 77]. Regardless of the types of vaccination routes, measuring mucosal IgA in saliva or feces, if any is detectable, helps evaluate vaccine responses, particularly in populations where blood collection is challenging.

Clinical studies consistently emphasize the significance of mucosal immune markers in evaluating the immunogenicity of norovirus vaccines [104]. High baseline and post-exposure levels of salivary and fecal IgA are closely linked to strong mucosal immune responses, leading to protection against norovirus gastroenteritis. Individuals with higher pre-existing salivary IgA or fecal IgA are more likely to have milder disease and reduced viral shedding following infection or challenge. Notably, studies on norovirus immunity in elderly populations in long-term care facilities demonstrated correlations between mucosal and serum IgA, and these immune responses are associated with protection against infection [105]. This prospective study demonstrated that norovirus-specific mucosal IgA is a sensitive marker of recent infection in the elderly. Salivary IgA levels rose rapidly by day 5 post-infection and remained elevated for at least three months. Significantly, salivary IgA correlated strongly with serum IgA titers and blockade antibodies (HBGA-blocking activity), underscoring its potential as a biomarker for both recent exposure and protective immunity.

Oral and intranasal norovirus vaccines administered via the mucosal route are more likely to activate mucosal-specific plasmablasts, driving localized IgA production at effector sites. The robust and durable mucosal IgA responses are expected to result in significant suppression of intestinal viral replication and reduced viral shedding [106]. For oral norovirus vaccine candidates, VP1-specific sIgA levels in saliva or in fecal samples provided direct evidence of mucosal immune activation in the gastrointestinal tract [107].

In infants, maternal transfer of norovirus-specific IgA via breast milk plays a crucial protective role. A birth cohort study in Peru found that higher titers and positivity rates of norovirus-specific IgA in breast milk were associated with a reduction in diarrheal symptoms in infected infants, emphasizing the importance of maternal mucosal immunity [108]. The study used ELISA assays with the VLPs representing diverse norovirus genotypes to quantify IgA responses. Interestingly, these studies provide a scientific basis for the concern that high maternal IgA in breast milk could interfere with the norovirus oral vaccine based on VLPs, similar to the observed interference with the live attenuated rotavirus oral vaccine through passive protection [109]. It has been shown that infants are born with maternal IgG antibodies, which decline rapidly to the lowest levels by approximately 5 months of age, which corresponds to the period when infants become more susceptible to natural norovirus infection [110] These findings suggest that vaccine clinical trial designs should carefully consider the timing of infant vaccination and optimize the booster schedule to account for the presence and waning of maternally acquired antibodies.

Taken together, measurements for antibodies in sera, saliva, and feces provide insights into a vaccine’s capacity to inhibit viral binding to host cell receptors and predict its clinical efficacy.

### Assessment of Neutralizing Antibody Titer

Neutralizing antibody (NAb) responses are a critical measure of norovirus vaccine efficacy, reflecting the ability of vaccine-induced antibodies to inhibit viral infection both *in vitro* and *in vivo*. Nab titers can be determined using HIE culture and patient fecal specimens carrying infectious noroviruses. More conveniently, HBGA- or PGM-blocking assays are often used as a surrogate assay for neutralizing antibody titrating.

### Surrogate Neutralization Antibody Titrating Assays

HBGA-blocking assay is a key method for evaluating the functional capacity of vaccine-induced antibodies to inhibit norovirus attachment to host cells, serving as a surrogate for virus neutralization [111, 112]. In this assay, synthetic HBGA oligosaccharides are used to determine the 50% blocking titer (BT_50_). The assay measures the ability of serum antibodies to block the binding of norovirus VLPs to HBGA receptors. For example, in a murine immunization study using chimeric rhesus rotavirus vectors expressing norovirus VP1 or P domain proteins, oral vaccination induced robust serum neutralizing antibody responses against the homologous GI.1 strain [113]. Similarly, HIL-214 (formerly TAK-214) VLP vaccine also generated substantial increases in HBGA-blocking antibody titers in vaccinated adults. In healthy adults aged 18 to 50 years who received two doses of VLP vaccine with adjuvants, a serum HBGA blocking antibody titer of >200 was shown to be associated with protection against illness and infection after challenge with a homologous virus [114].

These findings confirm that HBGA-blocking antibodies serve as surrogate markers for neutralizing activity and correlate strongly with protective immunity to norovirus infection [84].

In addition to the HBGA-blocking assays, a PGM-based neutralization assay is also widely employed [79]. PGM-blocking assay correlated well with other humoral responses in vaccinees receiving a bivalent VLP vaccine administered in the deltoid muscle [115].

### Cell-Based Neutralization Antibody Titrating Assays

Currently, no *bona fide* cell culture systems supporting all variants of HuNoVs have been established [54]. In addition, purified HuNoV from patient stool samples is generally not infectious in cell culture models [116], unlike other viruses. As such, the conventional plaque-forming assay cannot be applied for the quantification of neutralizing antibodies against the VP1 antigen.

Previously, various primary cells and established cell lines, such as the human B-lymphocyte-derived BJAB cell line, have been tested for their susceptibility to HuNoV infection [53, 117]. While viral RNA loads were found to increase to a certain level, as assessed by RT-qPCR, the fold increase in viral RNA copy number appears not to be sufficiently high for neutralizing assays using immunized sera. Despite its low replication efficiency, BJAB or primary immune cells supporting HuNoV still hold promise for further engineering via gene editing and the knock-in of pro-viral factors or the silencing of antiviral factors, targeting the cellular mechanisms involved in antiviral defense.

The meta-analysis suggested that individuals with the functional FUT2 gene were more vulnerable to norovirus infection [40]. Thus, a functional FUT2 gene (secretor phenotype), which expresses the HBGAs acting as an entry factor for HuNoV, is a feature to consider when selecting or optimizing NoV infection experiments.

In conjunction with the culture system, a promising approach that needs to be established is a norovirus replicon-based system, which can be used to generate diverse chimeric replicons expressing viral capsid proteins from variants by a reverse genetics approach [111, 118]. Yet, there is no infectious cDNA clone for norovirus available [54], in part due to the lack of a robust cell culture system established for HuNoV. When combined with HIE cultures, the replicon system can provide a physiologically relevant and applicable platform for cultivating diverse emerging NoV variants and assessing the cross-reactivity of immunized sera. This platform would be beneficial for assessing NAb responses, despite limitations such as lower replication efficiency, which have to be overcome.

It is anticipated that a generally accepted immunologic correlate of protection for norovirus vaccines will be defined in the future. Identifying and validating reliable immune markers that correlate with actual clinical protection remains a key research priority for advancing norovirus vaccine programs. In the meantime, in the absence of a conventional neutralization assay using a plaque or foci-forming assay using a live norovirus and a robust, convenient culture system, in addition to the yet-unknown receptor for the virus, blocking antibody titers determined by surrogate neutralization antibody titrating assays will be widely employed as surrogate immunologic endpoints [119, 120].

## Cellular Immune Responses to Norovirus Infection and Vaccination

While serum IgG/IgA and mucosal IgA serve as essential humoral immune response markers to monitor for vaccine efficacy assessment, another surrogate marker of immunity to consider is T cell immune responses. T cell responses enhance norovirus vaccine efficacy by targeting conserved viral antigenic epitopes across genotypes, mediating the clearance of infected cells, and are capable of contributing to the provision of long-term, cross-protective immunity [121].

Previous studies suggested that long-term mucosal immunity is maintained by memory T cells that retain their tissue-homing capabilities through epigenetic programming and dynamic migratory behavior [122, 123]. Gut-primed CD4^+^ memory T cells are epigenetically programmed to home to the intestinal mucosa, regardless of whether they are circulating in the blood or residing in peripheral tissues. Thus, following norovirus re-exposure or vaccination via oral or parenteral (*e.g.*, intramuscular) routes, these cells have the potential to coordinate the activation and differentiation of memory B cells, promoting robust mucosal antibody responses, including sIgA production. Concurrently, epigenetically imprinted CD8^+^ cytotoxic memory T cells patrol and reside within gut tissue, where they recognize and eliminate virus-infected enterocytes, thereby aiding local viral clearance.

The coordinated action of these long-lived, tissue-resident, and recirculating memory T cell populations ensures effective and rapid mucosal immune protection upon re-exposure. Thus, the effectiveness of norovirus vaccines is affected by the feature of how long-term mucosal immunity is maintained by memory T cells that retain their tissue-homing capabilities through epigenetic programming and dynamic migratory behavior.

As such, T-cell activation and cytokine production are critical for clearing infected cells and modulating adaptive immunity and can complement humoral responses by establishing long-term immune memory. Therefore, assessments of antigen-specific T-cell proliferation via flow cytometry and cytokine profiles (*e.g.*, IFN-γ, IL-2 ELISpot) provide insights into vaccine-induced cellular protection.

Standardized evaluation of these T cell responses is essential for understanding immune durability and cross-genotype efficacy, particularly against norovirus strains that evade antibody neutralization. This section reviews methods for evaluating T cell responses as critical correlates of immunity to norovirus vaccines, alongside assessments of mucosal and memory B cell responses.

### Assessment of Mucosal and Memory B Cell Responses as Correlates of Norovirus Vaccine-Induced Immunity

In addition to salivary and fecal IgA, which serve as biomarkers for strong mucosal immune responses, the expansion of norovirus-specific memory B cells is closely linked to protection against norovirus gastroenteritis [124]. After exposure to norovirus antigens (via vaccine or infection), B cells differentiate into antibody-secreting cells (ASCs), including plasmablasts and plasma cells, which produce specific antibodies. Some B cells develop into memory B cells, which do not secrete antibodies immediately but persist in the body and can rapidly become ASCs upon re-exposure, enabling a faster and stronger immune response [85].

Both IgA-secreting and IgG-secreting B cells contribute to long-term memory, with IgG memory B cells persisting in peripheral blood after infection [101]. Flow cytometric analysis has demonstrated that markers such as α4β7 are upregulated on memory B cells and plasmablasts induced by norovirus vaccines, consistent with their potential for mucosal homing [77]. In that study, bivalent VLP vaccination induced ASC responses that peaked at day 7 and then declined, while antigen-specific IgG memory B cells remained detectable for at least six months post-vaccination. Similarly, a tablet oral vaccine based on a nonreplicating adenovirus vector was shown to induce VP1-specific circulating ASCs in PBMCs and IgA^+^ memory B cells expressing the gut-homing receptor α4β7, in addition to elevating fecal IgA and salivary antibody titers [77]. Moreover, mucosal administration of a GII.4 P domain particle (Pd) vaccine via combined intranasal (IN) and sublingual (SL) routes, adjuvanted with *Vibrio vulnificus* flagellin (FlaB), promoted both humoral and cell-mediated immune responses as well as fecal sIgA production [125]. Together, these findings highlight that detection of vaccine-induced, gut-homing B cell responses in circulation can provide valuable insight into mucosal immunity, particularly when direct intestinal sampling is impractical in clinical studies.

Nevertheless, evidence from a murine norovirus model indicates that effective viral clearance and durable mucosal immunity require a coordinated response from both CD4^+^ and CD8^+^ T cells in addition to B cells, with T cell cytotoxic pathways, particularly perforin-mediated killing, playing a central role [126, 127]. Consistent with these mechanistic insights, Vaxart’s oral tablet norovirus vaccines have demonstrated the ability to elicit mucosal immunity and reduce viral shedding in Phase 2 challenge studies [128]. Altogether, profiling salivary/fecal IgA, gut-homing memory B cells, and coordinated T cell responses provides critical biomarkers of vaccine efficacy and correlates of protection against norovirus [101].

In summary, assessing antibody-secreting cell frequencies, antigen-specific memory B cells, and T cell function is crucial for evaluating norovirus vaccine effectiveness and for gaining a better understanding of the correlates of protection. In particular, quantification of antigen-specific IgA and α4β7-expressing, gut-selective B cells in the circulation is critical, besides the measurement of sIgA in saliva and/or feces.

### Assessment of Cellular Immune Responses to Norovirus Vaccines through T Cell Assays

A perforin-dependent mechanism for CD8^+^ T-cell-mediated viral clearance in the intestinal epithelium is strongly supported by studies utilizing murine norovirus infection models. In these studies, norovirus-specific CD8^+^ T cells were shown to infiltrate the intestinal lamina propria to target infected cells. Adoptive transfer experiments demonstrated that perforin, a pore-forming protein essential for CD8^+^ T cell cytotoxicity, is critical for reducing viral loads in the intestine [121].

To evaluate such vaccine-induced cellular immunity in humans and animal models, several functional T cell assays are employed. Intracellular cytokine staining (ICS) is used to profile multifunctional antigen-specific CD4^+^ T cells by detecting co-expression of IFN-γ, interleukin-2 (IL-2), and tumor necrosis factor-alpha (TNF-α) after ex vivo stimulation with vaccine antigens such as VLPs or peptides. ICS is a gold-standard assay for detailed functional characterization of vaccine-induced T cell responses in PBMC samples [115]. In a clinical trial of healthy adults vaccinated intramuscularly with a bivalent norovirus vaccine candidate based on VLPs of genotypes GI.4 and GII.4, ICS performed on PBMCs revealed antigen-specific CD4^+^ T cell responses, but a CD8^+^ T cell response, typical of natural infection, was not detected [115]. In contrast, in a murine model of norovirus mRNA-LNP vaccination, multiparametric flow cytometry demonstrated the induction of both antigen-specific CD4^+^ and CD8^+^ T cells, with CD8^+^ T cells producing predominantly IFN-γ and TNF-α, consistent with a multifunctional effector phenotype [79].

The ELISpot assay provides a sensitive, complementary approach for detecting IFN-γ–secreting T cells in response to norovirus antigens. Using PBMCs from healthy adult donors, ELISpot confirmed antigen-specific cellular responses to VLP vaccination, although this particular study (*n* = 8) did not observe a correlation between pre-existing antibody levels and T cell activity [129].

T cell proliferation can also be evaluated by measuring antigen-driven lymphocyte expansion. Traditionally, this has been done using ^3^H-thymidine incorporation, which quantifies DNA synthesis during cell division [73]. In a Phase I trial of a plant-produced VLP vaccine, stimulation indices (antigen-stimulated vs. control cultures) increased from day 1 to day 57, before gradually declining by day 365, suggesting transient but measurable cell-mediated immunity. A more refined tool, the carboxyfluorescein succinimidyl ester (CFSE) dilution assay, employs flow cytometric measurement of fluorescence dilution with each cell division, thereby enabling precise quantification of antigen-specific proliferating T cells [130].

## Vaccine Administration Routes and Adjuvant Effects

In addition to conventional assessments of humoral and cellular immune responses, it is essential to consider the influence of adjuvants and routes of immunization. These factors profoundly affect the magnitude, quality, and localization of immune responses, thereby playing a critical role in optimizing protection against infectious diseases.

### Systemic and Mucosal Immune Responses Elicited by Norovirus Vaccines Administered via Parental or Mucosal Routes

**Fig. 3:**
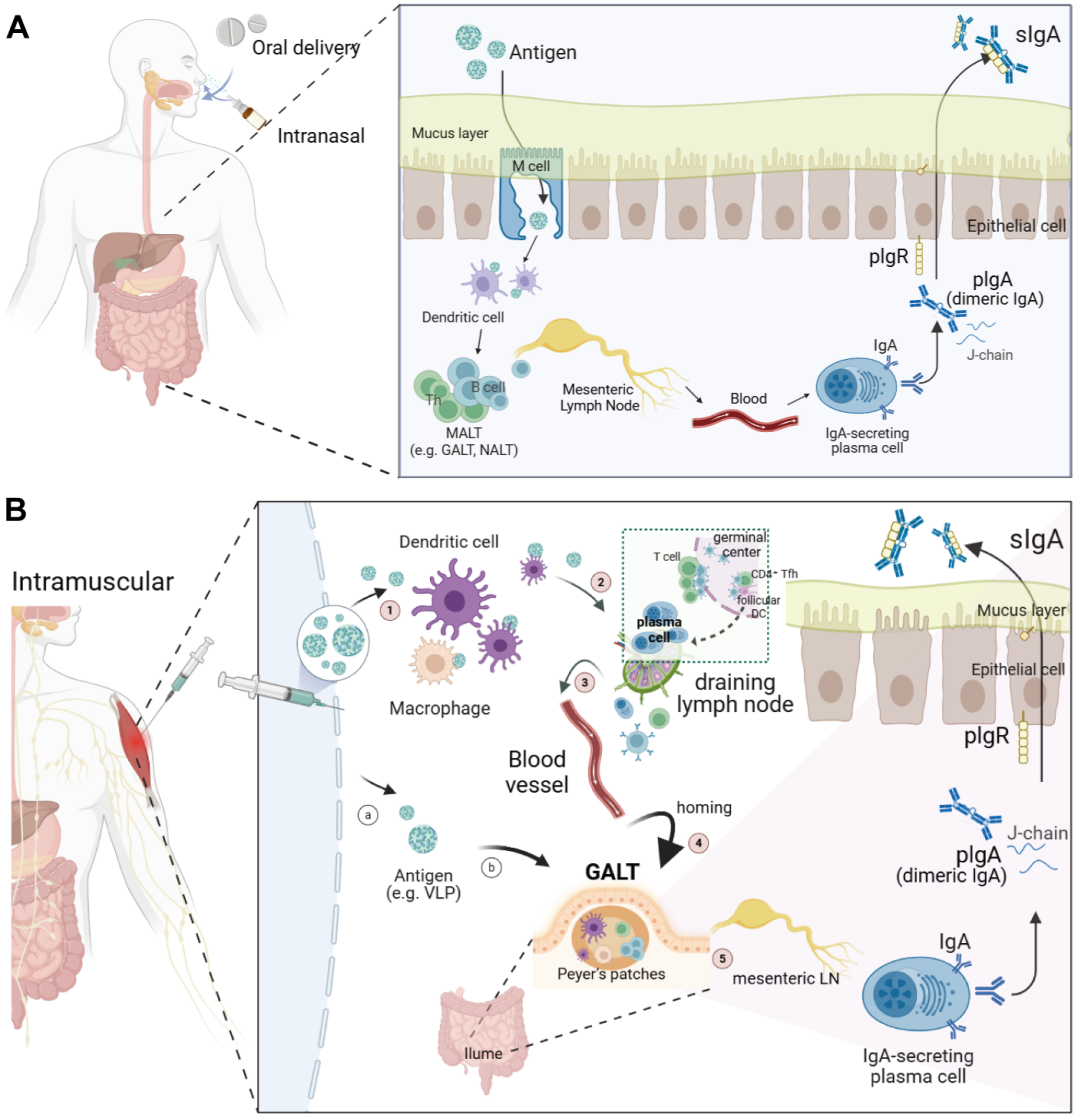
Pathways and mechanisms of mucosal sIgA induction by HuNoV vaccine administered via oral, intranasal, or intramuscular routes. (**A**) Mucosal (oral, intranasal) vaccine routes and natural infection. sIgA responses are efficiently elicited at mucosal surfaces by natural infection and administration of norovirus antigens through oral or intranasal routes. These routes primarily stimulate antigen uptake and B cell activation within mucosa-associated lymphoid tissues (MALT), such as gut-associated lymphoid tissue (GALT) and nasopharynx-associated lymphoid tissue (NALT), resulting in the differentiation and local accumulation of IgA-secreting plasma cells in the intestinal lamina propria, thereby producing sIgA at the site of viral entry. (**B**) Proposed mechanisms for mucosal sIgA induction by IM vaccine administration. One potential pathways involved: (1) antigen uptake by APCs [dendritic (DC) cells and macrophages] at the injection site, (2) their migration to draining lymph nodes, and subsequent activation of T follicular helper (Tfh) cells and B cells, or alternatively mRNA-LNP traffics to the lymph nodes to prime APCs and Ag-primed B cells [132], (3) differentiation of activated B cells into IgA-committed plasmablasts expressing the mucosal-homing integrin α4β7, (4) migration of these α4β7^+^ plasmablasts through systemic circulation by entering the bloodstream to mucosal tissues, including the intestinal lamina propria, along with the activated Tfh as well as trafficking of antigen-specific CD8^+^ T cells to mucosal compartment [133], and (5) local differentiation into IgA-secreting plasma cells, resulting in sIgA production at norovirus entry sites in the gut epithelium. Alternative pathways include: (a) trafficking of the antigens (*e.g.*, VLPs) or antigen-exposed APCs at the injection site to MALT, including GALT and NALT, (b) activation of local immune responses within MALT compartments.

The choice of vaccine platform and administration route significantly affects both the type and magnitude of systemic and mucosal immune responses elicited by norovirus vaccines. Mucosal delivery of norovirus antigens (via oral or intranasal routes) or natural infection can directly target MALT inductive sites, more efficiently inducing robust gut sIgA responses and establishing durable adaptive mucosal immunity for neutralizing norovirus at its primary portal of entry, thereby blocking initial infection and subsequent viral dissemination (Fig. 3A). The migration of α4β7^+^CCR9^+^ IgA-committed B cells to mucosa-associated lymphoid tissues (MALT) for local sIgA production at the intestinal epithelial surface represents the primary protective mechanism against noroviral infection.

Unlike adenovirus norovirus oral vaccines, current VLP-based vaccines administered intramuscularly have limitations in inducing durable mucosal immunity in the gut, while they generate robust systemic humoral responses [68]. Likewise, a critical unresolved question in vaccine immunology is whether IM administration of mRNA vaccines can effectively elicit mucosal immune responses. As illustrated in Fig. 3B, IM administration of an mRNA vaccine can induce robust and durable systemic immune responses, primarily within secondary lymphoid organs such as the germinal centers (GCs) of draining lymph nodes. In these sites, B cells undergo affinity maturation and differentiate into memory B cells and long-lived plasma cells. While systemic immunity induced by intramuscular mRNA vaccines is well documented, the extent to which these vaccines generate robust mucosal IgA production and promote mucosal tissue-resident memory lymphocytes remains uncertain.

Recent studies indicate that COVID-19 mRNA vaccines can induce mucosal antibody response, particularly in individuals with prior SARS-CoV-2 exposure [100]. Moreover, IM COVID-19 mRNA vaccination elicited intranasal mucosal responses, including neutralizing IgG and IgA in nasal epithelial lining fluid [131]. Mechanistically, it might be possible that a fraction of memory B and T cells generated in GCs upregulate mucosal-homing receptors, including integrin α4β7 and CCR9, enabling their migration from the circulation to mucosa-associated lymphoid tissues (MALT) such as the nasopharynx-associated lymphoid tissue (NALT) and the gut-associated lymphoid tissue (GALT). Upon re-exposure to antigen, either through booster vaccination or natural infection, these mucosal-homed memory cells in the NALT or GALT can be rapidly reactivated. Alternatively, antigen or antigen-exposed antigen-presenting cells (APCs) trafficking to the GALT may activate local immune responses. Moreover, in populations with pre-existing norovirus exposure, memory recall responses may enhance vaccine antigen recognition and accelerate sIgA responses by rapidly activating memory B cells that express mucosal-homing receptors. The last possible pathway involves the trafficking of circulating antigen-primed, memory B/T cells from the GALT or secondary lymphoid organs to the draining lymph nodes, where they are reactivated by the antigen or primed APCs and then home to the GALT (not indicated in the figure).

These potential mechanistic scenarios help explain how IM mRNA vaccination can generate systemic immunity that is functionally extended to mucosal sites, thereby bridging systemic and mucosal immune compartments. However, whether a similarly administered norovirus mRNA vaccine would elicit mucosal sIgA in naïve or seronegative individuals remains uncertain.

In summary, systemic IgG titers serve as the primary marker of response following parenteral vaccination, whereas mucosal IgA titers in secretions such as saliva or feces reflect mucosal immunity [134, 135]. Systemic vaccination, including IM or SC delivery of mRNA or VLP vaccines formulated with a proper adjuvant, may induce modest mucosal IgA responses. However, these responses are generally less robust compared to direct mucosal immunization routes such as IN or oral delivery, which target inductive MALT sites and elicit stronger local sIgA production. Despite the challenge of achieving strong mucosal immunity through parenteral routes, eliciting such responses remains a critical goal for vaccines targeting mucosal pathogens, including noroviruses. By contrast, while mucosal routes (oral, intranasal) are highly effective in inducing local immunity, they face challenges such as safety concerns (*e.g.*, Bell’s palsy in intranasal vaccines) and physiological barriers (*e.g.*, gastric acid degradation in the stomach, oral tolerance). These limitations underscore the continued need for innovative parenteral vaccine strategies that can safely and effectively induce robust mucosal immunity.

### Adjuvants: Simple Immune Boosters or Mucosal Immune Response Boosters?

Several adjuvants approved for human vaccines, which include MF59 (oil-in-water emulsion), the AS0 Adjuvant Systems, and CpG 1018, can enhance norovirus vaccine efficacy by stimulating cytokine production and T/B cell activation [136]. *In vitro* screening using PBMCs from healthy donors can help identify promising adjuvant candidates while minimizing confounding effects from pre-existing immunity [137].

In a preclinical mouse study of a norovirus GII.4 VLP vaccine, adjuvants including AS04, composed of an aluminum salt plus monophosphoryl lipid A (MPLA), a TLR4 agonist, significantly enhanced binding and functional antibody responses [138]. While aluminum hydroxide Al(OH)3 or MPLA adjuvants induced systemic Th2-skewed antibody profiles, they did not enhance mucosal IgA production [139]. Interestingly, IN delivery of the same VLP vaccine without an adjuvant induced both systemic and mucosal IgA antibody responses, demonstrated by increased antibody levels in fecal and nasal samples, suggesting that mucosal delivery itself can effectively prime mucosal immunity without requiring an adjuvant.

Moreover, some adjuvants have been reported to promote mucosal immune responses even following parenteral immunization. For example, the second-generation lipid adjuvant in stable emulsion (SLA-SE), a synthetic hexa-acylated lipid A formulated in a stable oil-in-water emulsion with an optimized TLR4 agonist, enhanced mucosal antibody responses after IM immunization with a recombinant enterotoxigenic *E. coli* (ETEC) vaccine antigen [140]. TLR ligands such as TLR3 and TLR4 agonists can modulate DC migration patterns, facilitating mucosal responses by directing DCs to both draining and nondraining lymph nodes [141, 142]. Although not yet evaluated in norovirus vaccines, natural and synthetic TLR4 agonists such as poly-γ-glutamic acid and neoagarose (a galacto-oligosaccharide derived from agar) hold promise as mucosal adjuvants [139, 143, 144].

Recently, LNPs, as used in mRNA vaccines, have been proposed to act as potent adjuvants for VLP-based norovirus vaccines by markedly increasing antibody titers [145]. The ionizable lipids in mRNA-LNP COVID-19 vaccines activate innate immunity through the TLR4 pathway [146]. Furthermore, mRNA nucleoside modifications influence TLR engagement; for instance, N1-methylpseudouridine dampens TLR3 activation [147]. Incorporating unmodified uridine may boost mucosal immune responses through TLR3 stimulation but could also reduce antigen expression due to type I interferon induction [148]. Importantly, norovirus VLPs themselves bind and activate TLR2 and TLR5 [149], suggesting that innate immune signaling through these receptors may also contribute to vaccine immunogenicity. Collectively, leveraging TLR-targeting adjuvants and optimized ionizable lipids in non-modified mRNA-LNP norovirus vaccines holds promise for eliciting robust mucosal immunity.

## Gaps between the Vaccine Preclinical Studies and Real-World Studies

Significant discrepancies exist between norovirus vaccine-induced immune responses observed in preclinical models and those seen in real-world human studies. These differences are particularly noticeable in the durability and quality of serum IgA and IgG, mucosal sIgA, memory B cell responses, and the extent of cross-protective immunity influenced by individuals’ prior norovirus exposures [150].

Norovirus vaccines target vulnerable populations such as infants and the elderly, where factors like pre-existing immunity, mucosal immune competence, and exposure history can significantly affect vaccine efficacy. For example, a seroepidemiological study of 386 serum samples from diverse age groups in South Korea revealed early childhood (2–5 years) seropositivity to norovirus genotypes GI.4, GII.3, and most notably, the globally dominant GII.4, for which IgG seroprevalence exceeded 94.5%, indicating widespread and early-life exposure [151].

GII.4 variants account for the majority of norovirus infections worldwide, and individuals commonly experience repeated infections with diverse GII.4 strains throughout their lifetime. Consequently, baseline immunity and memory B cell repertoires in most populations are skewed heavily towards GII.4. In such pre-exposed populations, a single norovirus vaccine dose often robustly boosts immune responses via memory recall, particularly in older children and adults [114, 150]. This epidemiological reality raises important concerns about immune imprinting, whereby pre-existing immunity to GII.4 may limit the breadth of vaccine-induced protection. Individuals with extensive GII.4 exposure or those in endemic regions may preferentially boost existing GII.4 memory responses upon vaccination, potentially limiting balanced immunity against other genotypes. Even multivalent vaccines may be influenced by this immunodominance phenomenon, reducing the effectiveness of expanded-valency formulations.

To overcome this, vaccine strategies may need to be designed with booster regimens targeting non-GII.4 strains to promote broader immunity. Without addressing immune imprinting, multivalent vaccines may risk reinforcing existing immunity at the expense of balanced protection. Furthermore, increasing the duration of antigen expression is also a plausible strategic approach to strengthen B cell maturation, yielding higher-avidity, more durable, and potentially broader antibody responses, which are critical for effective norovirus vaccines. Extending antigen expression, such as through self-amplifying RNA platforms, optimized mRNA stabilization, or enhancing antigen expression by optimizing an mRNA platform, can help overcome the limitations posed by immune imprinting by driving more robust B cell maturation against diverse epitopes [152, 153]. This may broaden the antibody repertoire and reduce immunodominance effects, leading to enhanced cross-reactive protection against multiple genotypes. Furthermore, the cross-reactivity of systemic serum IgG and fecal secretory IgA across diverse norovirus variants should be systematically evaluated to assess the breadth of vaccine-induced immunity, as the cross-reactivity of these key immune correlates, particularly sIgA, is crucial for defense at the entry site and has not yet been thoroughly investigated in preclinical or clinical studies.

Addressing these challenges requires comprehensive immunological data from pre-exposed populations to understand the quality, breadth, and longevity of vaccine-induced responses. Such data are critical for guiding vaccine design and optimizing immunization schedules, ensuring broad and durable protection against evolving norovirus strains.

## Conclusion

The development of effective human norovirus vaccines has been hindered by several significant challenges, including the high genetic diversity of noroviruses, the absence of robust *in vitro* culture systems and suitable animal models, and the lack of well-validated immune correlates of protection. Despite these hurdles, substantial progress has been made through the advancement of various vaccine platforms, including VLP, mRNA, and viral vector-based candidates.

Numerous vaccine candidates have demonstrated encouraging immunogenicity and efficacy in both preclinical and clinical studies. Moreover, innovative tools, including human intestinal organoid models and standardized neutralization assays, are improving the assessment of vaccine-induced immune responses and cross-protective potential.

Despite these advances, key challenges remain. Critical among them is the need to establish standardized efficacy endpoints and to validate broad protective immunity effective against the diverse norovirus strains circulating worldwide. Addressing these gaps is essential to advancing the development of effective norovirus vaccines.

A comprehensive assessment of vaccine efficacy should integrate multiple immunological parameters, including neutralizing antibody titers, HBGA-blocking activity, mucosal IgA responses, T cell-mediated immunity, and B cell responses, encompassing the induction and persistence of antigen-specific memory B cells and antibody-secreting cells. Emerging evidence indicates that robust B cell memory, particularly IgG and IgA memory B cells, can persist for months after vaccination with VLP vaccines and correlates with sustained protective immunity, potentially serving as additional correlates of protection. Such multifaceted immunological profiling is crucial for fully characterizing protective immunity and guiding the rational design and rigorous evaluation of broadly protective norovirus vaccines.

## Figures and Tables

**Table 1:** Major human norovirus vaccine candidates.

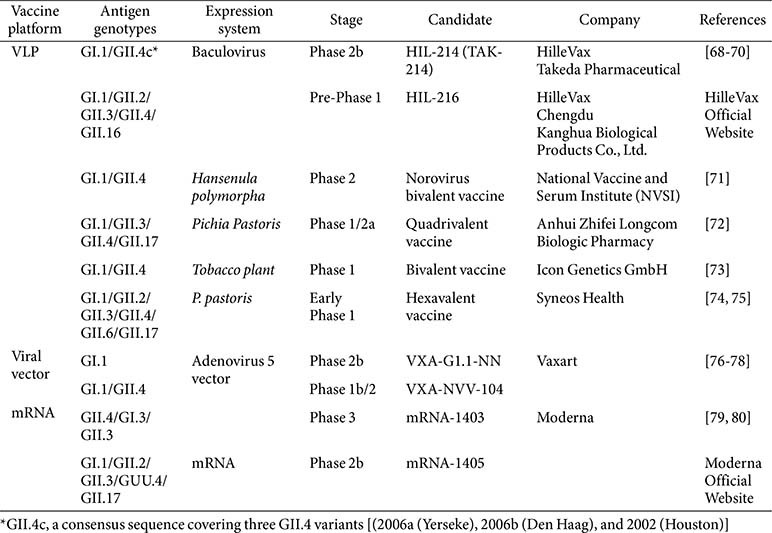

**Table 2:** Representative HuNoV vaccines: assays used to evaluate their immune responses in preclinical and clinical studies.